# Supplementary material for: Comparison of Gut Bacterial Communities of Locusta migratoria manilensis (Meyen) Reared on Different Food Plants
Source: Biology (Basel). 2022 Sep 13;11(9):1347. doi: 10.3390/biology11091347 (PMC9495404; doi:10.3390/biology11091347)
Supplement: Supplementary file 1 [file biology-11-01347-s001.zip › biology-1882540-Supplementary Materials/Supplementary Figure S1.pdf]

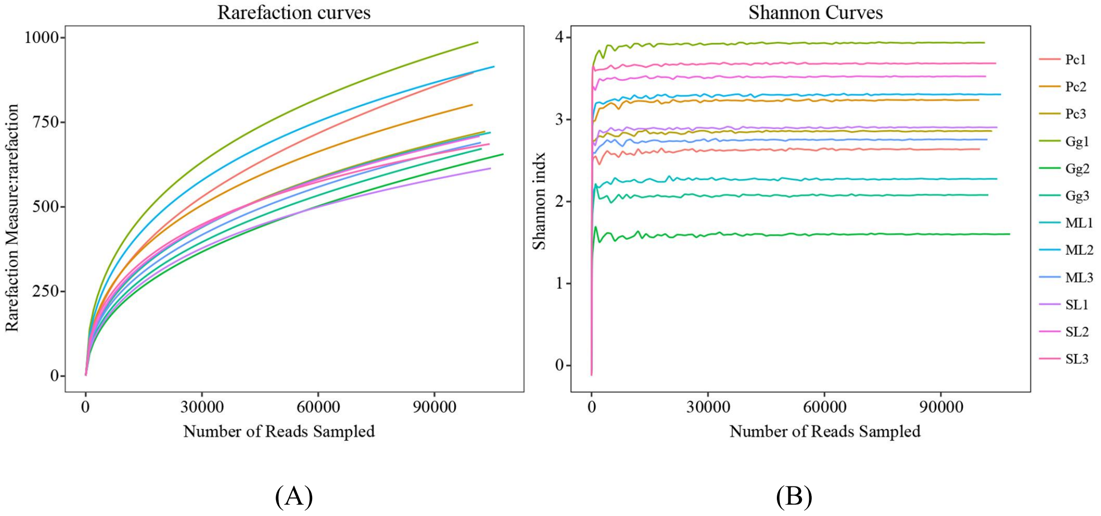


**Figure S1. (A) Sample rarefaction curves and (B) Shannon index rarefaction curves of individual samples for metagenomic analysis of gut microbiota of *Locusta migratoria manilensis* fed on four different food plants.** ML: maize leaves; Gg: goosegrass; SL: soybean leaves; Pc: pakchoi.
