# Supplementary material for: Comparison of Gut Bacterial Communities of Locusta migratoria manilensis (Meyen) Reared on Different Food Plants
Source: Biology (Basel). 2022 Sep 13;11(9):1347. doi: 10.3390/biology11091347 (PMC9495404; doi:10.3390/biology11091347)
Supplement: Supplementary file 1 [file biology-11-01347-s001.zip › biology-1882540-Supplementary Materials/Supplementary Figure S2.pdf]

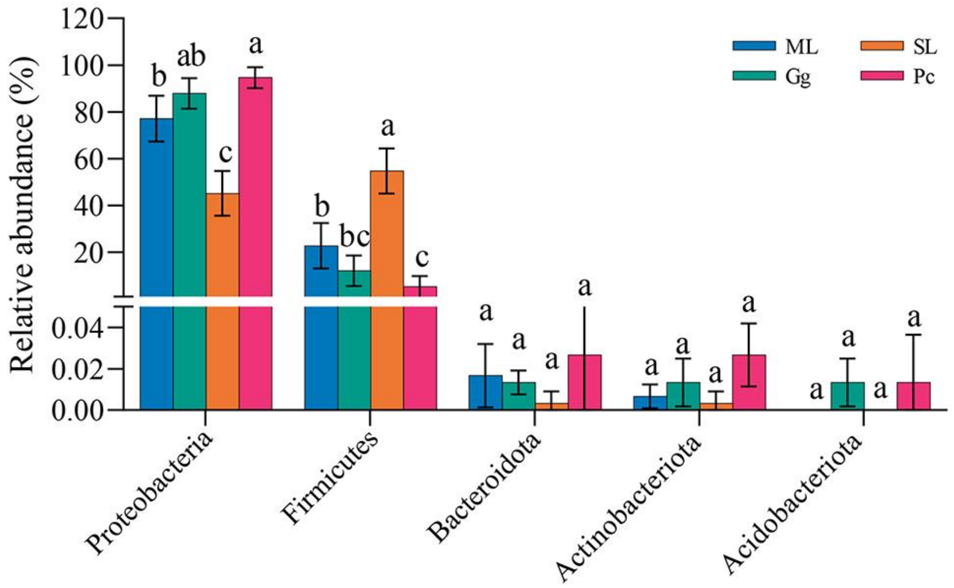

Figure S2. The top 5 relative abundances of bacteria at the phylum level in the gut of *Locusta migratoria manilensis* fed on four different food plants. ML: maize leaves; Gg: goosegrass; SL: soybean leaves; Pc: pakchoi. Bars marked by different lowercase letters are significantly different based on Turkey's HSD analysis at P＜0.05.
