# Supplementary material for: Comparison of Gut Bacterial Communities of Locusta migratoria manilensis (Meyen) Reared on Different Food Plants
Source: Biology (Basel). 2022 Sep 13;11(9):1347. doi: 10.3390/biology11091347 (PMC9495404; doi:10.3390/biology11091347)
Supplement: Supplementary file 1 [file biology-11-01347-s001.zip › biology-1882540-Supplementary Materials/Supplementary Figure S3.pdf]

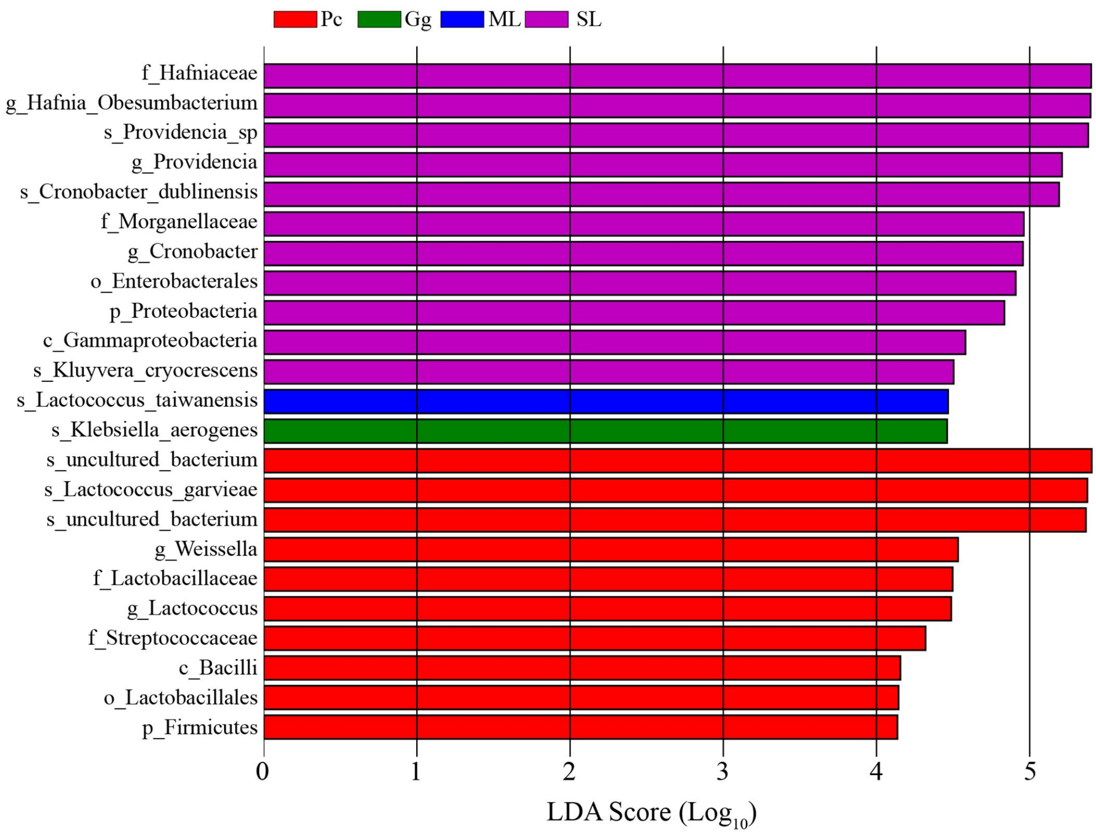

Figure S3. Histogram of LDA value distribution of the gut bacterial community of *Locusta migratoria manilensis* fed on different food plants. ML: maize leaves; Gg: goosegrass; SL: soybean leaves; Pc: pakchoi. The ordinate is the taxon with significant differences between groups, while the abscissa is a bar chart that visually displays the LDA difference analysis logarithm score of the corresponding taxon, and sorts them according to the score value, so as to describe their differences in different group samples. The longer the length, the more significant the difference of the taxon, and the different colors of the bar chart indicate the group of samples with higher abundance corresponding to the taxon.
