# Supplementary material for: Comparison of Gut Bacterial Communities of Locusta migratoria manilensis (Meyen) Reared on Different Food Plants
Source: Biology (Basel). 2022 Sep 13;11(9):1347. doi: 10.3390/biology11091347 (PMC9495404; doi:10.3390/biology11091347)
Supplement: Supplementary file 1 [file biology-11-01347-s001.zip › biology-1882540-Supplementary Materials/Supplementary Table S1.pdf]

Table S1. Summary of high-throughput sequencing statistics obtained for each sample evaluated in the present study.

Sample ID	Raw reads	Clean reads	Effective reads	AvgLen (bp)	GC (%)	Effective (%)
Ma1	107096	107086	104526	477	54.20	97.60
Ma2	107705	107687	105482	477	53.78	97.94
Ma3	102341	102330	101997	477	53.45	99.66
Gg1	103479	103462	101319	477	53.90	97.91
Gg2	109241	109227	107829	477	54.60	98.71
Gg3	104496	104485	102215	477	53.87	97.82
Sb1	107840	107806	104571	477	52.46	96.97
Sb2	103788	103769	101632	477	53.25	97.92
Sb3	106675	106652	104240	477	53.07	97.72
Cc1	106511	106505	100088	477	54.68	93.97
Cc2	102145	102131	99879	477	55.07	97.78
Cc3	104643	104637	103106	477	54.98	98.53

Average length (bp) is the average sequence length of all samples. GC (%) is the percentage of G and C type bases in the total base. Effective (%) is the percentage of effective reads in raw reads.
