# Supplementary material for: Comparison of Gut Bacterial Communities of Locusta migratoria manilensis (Meyen) Reared on Different Food Plants
Source: Biology (Basel). 2022 Sep 13;11(9):1347. doi: 10.3390/biology11091347 (PMC9495404; doi:10.3390/biology11091347)
Supplement: Supplementary file 1 [file biology-11-01347-s001.zip › biology-1882540-Supplementary Materials/Supplementary Table S2.pdf]

Table S2. The top 10 relative abundances at the family level in *Locusta migratoria manilensis* fed on four different food plants.

Taxonomy	Maize leaves	Goosegrass	Soybean leaves	Pakchoi
Enterobacteriaceae	75.88±9.106^{a}	85.92±7.005^{a}	44.79±9.690^{b}	84.37±5.735^{a}
Streptococcaceae	9.38±4.324^{b}	4.20±1.625^{b}	35.56±9.110^{a}	4.72±4.442^{b}
Lactobacillaceae	13.4±6.846^{ab}	7.44±4.662^{bc}	18.85±1.280^{a}	0.16±0.100^{c}
Morganellaceae	0.26±0.224^{a}	0.57±0.462^{a}	0.03±0.030^{a}	6.12±6.292^{a}
Hafniaceae	0.41±0.504^{a}	0.56±0.504^{a}	0.00±0.010^{a}	2.77±2.362^{a}
Erwiniaceae	0.41±0.311^{a}	0.56±0.655^{a}	0.34±0.200^{a}	0.18±0.108^{a}
Enterococcaceae	0.01±0.012^{a}	0.43±0.561^{a}	0.39±0.300^{a}	0.22±0.198^{a}
Moraxellaceae	0.08±0.040^{ab}	0.03±0.029^{b}	0.00±0.010^{b}	0.48±0.297^{a}
Yersiniaceae	0.00±0.006^{a}	0.05±0.061^{a}	0.00±0.010^{a}	0.53±0.777^{a}
Pseudomonadaceae	0.09±0.061^{a}	0.14±0.211^{a}	0.00±0.010^{a}	0.07±0.017^{a}

Data are reported as the mean ± SE. Different lowercase letters (a, b, c) indicate significant differences in the mean values of relative abundance within the same line. Same lowercase letters indicate no significant difference between sample groups. Different lowercase letters indicate significant differences in the mean values among sample groups (One-way ANOVA, Tukey post-hoc test, $p < 0.05$).
